# A Review on Finite Element Modeling and Simulation of the Anterior Cruciate Ligament Reconstruction

**DOI:** 10.3389/fbioe.2020.00967

**Published:** 2020-08-20

**Authors:** Lefteris Benos, Dimitar Stanev, Leonidas Spyrou, Konstantinos Moustakas, Dimitrios E. Tsaopoulos

**Affiliations:** ^1^Institute for Bio-Economy and Agri-Technology, Centre for Research and Technology-Hellas, Thessaloniki, Greece; ^2^Department of Electrical and Computer Engineering, University of Patras, Patras, Greece; ^3^School of Engineering, Institute of Bioengineering, École Polytechnique Fédérale de Lausanne, Lausanne, Switzerland

**Keywords:** ACL reconstruction, finite element analysis, graft modeling, graft type, graft fixation, graft pretension

## Abstract

The anterior cruciate ligament (ACL) constitutes one of the most important stabilizing tissues of the knee joint whose rapture is very prevalent. ACL reconstruction (ACLR) from a graft is a surgery which yields the best outcome. Taking into account the complicated nature of this operation and the high cost of experiments, finite element (FE) simulations can become a valuable tool for evaluating the surgery in a pre-clinical setting. The present study summarizes, for the first time, the current advancement in ACLR in both clinical and computational level. It also emphasizes on the material modeling and properties of the most popular grafts as well as modeling of different surgery techniques. It can be concluded that more effort is needed to be put toward more realistic simulation of the surgery, including also the use of two bundles for graft representation, graft pretension and artificial grafts. Furthermore, muscles and synovial fluid need to be included, while patellofemoral joint is an important bone that is rarely used. More realistic models are also required for soft tissues, as most articles used isotropic linear elastic models and springs. In summary, accurate and realistic FE analysis in conjunction with multidisciplinary collaboration could contribute to ACLR improvement provided that several important aspects are carefully considered.

## Introduction

Knee is one of the most elaborate joints in the human body. It is subject to large loads and, as a consequence, prone to injury. In particular, knee is classified as the most regularly injured joint in athletes, since participation in sports activities involves high tissue loading accompanied with high pivoting. Among knee injuries, anterior cruciate ligament (ACL) rupture is the most common one and causes anteroposterior laxity leading to an unstable knee (Legnani et al., 2010). Predisposing factors include biomechanical and neuromuscular abnormalities, sex hormones, mutations of collagen producing genes and structural influences of the knee (Vaishya et al., 2015).

Anterior cruciate ligament has a very poor healing capacity, which has been corroborated by a number of *in vivo* and *in vitro* experiments (Mahapatra et al., 2018). Furthermore, no local healing takes place in complete ruptures (Hefti et al., 1991). The formation of a fibrin-platelet scaffold, which would contribute to the primary ACL healing, is prevented by the intra-articular movement as well as the synovial fluid (Murray et al., 2010). In persons with ACL-deficient knees, knee instability is observed, while other components of the joint are also at risk of injury, such as menisci and cartilages (Mehl et al., 2019).

The inability of ACL to regenerate after injury is the primary reason why ACL reconstruction (ACLR) is regarded as the gold standard treatment (Mahapatra et al., 2018). During this surgical operation, a graft is employed to replace the injured ligament. Therefore, it should be clarified that ACLR, which is the subject of this study, is a completely different procedure than the ACL repair. The latter aims at preserving and repairing the native tissue (Mahapatra et al., 2018). Although ACLR has been established as the preferred choice, there is a plethora of different surgery approaches depending on the surgeon’s experience as well as the patient’s condition.

For example, the tunnel in femur can be accomplished either via a transtibial or anteromedial technique. The latter is also known as transportal technique. The transtibial technique utilizes the graft in a relatively vertical position, whereas the anteromedial technique enables the surgeon to choose the femoral tunnel position according to the patient’s needs (Vaishya et al., 2015). Non-anatomic and anteriorly located femoral tunnels can be observed with the transtibial technique, while the anteromedial technique results in an anatomic ACLR (Arno et al., 2016). Which of the two approaches leads to better clinical outcome is still a topic of debate (Chen et al., 2017). Some researchers reported that the anteromedial technique gives better functional outcomes and knee stability (Mandal et al., 2012; Brown et al., 2013), but on the other hand, there is plenty of support that transtibial technique has similar outcomes (Arno et al., 2016; Ozel et al., 2017). Apart from the different techniques pertaining to the femoral tunnel, there are various approaches regarding the grafts used as substitutes of the original ACL, operative techniques, fixation devices and initial graft tension (or pretension), which will be presented in this study.

Computational biomechanics furnishes a new methodology that can offer beneficial information that are hard to be obtained experimentally (Stanev et al., 2016). Besides, *in vivo* experiments are very costly, time-consuming and technically complicated (Weiss et al., 1996). However, development of accurate numerical models of the knee joint is a demanding task, mainly on account of the intricate nature of the joint itself and the realistic mechanical properties that should be assigned to the soft tissues. Finite element (FE) methodology is an ideal tool for capturing the effect of geometry and material properties on the mechanics of the knee. Subject-specific geometries can be obtained through magnetic resonance (MR) imaging and discretized into FE. Subsequently, material models and properties are assigned to the knee components, while adequate initial and boundary conditions as well as interactions between them are imposed. With FE simulations one can test hypotheses and understand the complex cause-effect of different loading conditions and the response of the soft tissues.

A validated numerical model can facilitate the evaluation of the overall effect of a plethora of variables related to graft tension, tunnel dimensions and selection of graft. It can also assess alternative techniques for the ACLR, that otherwise would require a considerable number of patients and the design of complex experimental setups (Richter et al., 2018). As a consequence, FE analysis can contribute not only to decrease the cost of treatment, but also to the optimization of current methodologies and investigation of new ones. Current progress in ACLR has been summarized in review papers such as Fu et al. (1999), Beasley et al. (2005), Duquin et al. (2009), Kim et al. (2013), and Vaishya et al. (2015). However, to the best of our knowledge, the progress in numerical modeling, as assistive tools for pre-surgery planning, has not been reported in the literature. The present review focuses on providing a systematic investigation of what has been achieved in FE simulation of ACLR so far along with discussing the up-to-date advancement in this operation.

Hereafter, this manuscript is divided into four sections. In section “Methods,” the search methodology is described in conjunction with the imposed exclusion criteria. Subsequently, an extensive section pertaining to the “Finite Element Modeling of ACLR Reconstruction” follows, which includes five subsections, namely the “Knee Joint Anatomy,” “Geometry Modeling and Mesh Generation,” “Loading and Boundary Conditions,” “Material Modeling and Properties of Grafts” and “Verification and Validation Assessment.” Finally, an analysis on “Simulation of Different Surgery Techniques” is also presented along with the current progress in each treatment, where the main differentiation of the chosen studies was observed. These techniques include “Single- or Double-Bundle ACL Reconstruction,” “Graft Options,” “Graft Fixation,” and “Graft Pretension.” This review ends with the section “Conclusion” with the intention of highlighting the most important results, suggesting future directions as well as stating the “Study Limitations and Strengths.”

## Methods

**FIGURE 1:**
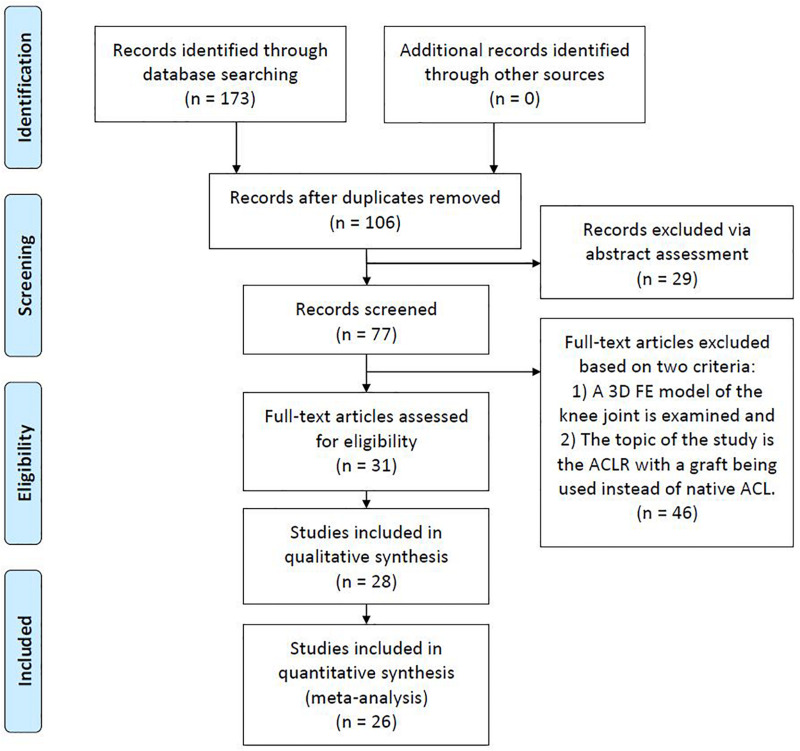
Flowchart of the present review methodology.

The search engines of Google Scholar, PubMed, and Scopus were used with the object of finding publications related to FE modeling and simulation of ACLR. For this purpose, different keyword combinations of “finite element,” “ACL reconstruction” and “knee joint biomechanics” were used. The date of the last search was February 28, 2020. Based on their title and abstract, the papers were filtered in order to select those ones that meet the following two basic criteria: (a) a three-dimensional (3D) FE model of the knee joint is examined and (b) the topic of the study is the ACLR with a graft being used instead of native ACL. Moreover, there is a plethora of studies investigating FE knee simulation using the material properties of an intact ACL. On the contrary, only 26 publications were found to meet the aforementioned criteria, mainly due to the versatile nature of the numerical problem itself and the theoretical background needed pertaining to the surgical operation. Furthermore, non-English articles, Master and Doctoral Theses were excluded from the aforementioned research. Figure 1, which is based on the PRISMA guidelines (PRISMA, 2020), shows a flowchart of the present methodology, while Table 1 includes all the selected publications along with a brief description of their main objective and followed validation method in a chronological order.

**TABLE 1:** List of papers dealing with the FE modeling and simulation of ACLR along with the methodology followed for validation assessment.

References	Main objective	Model validation method
Suggs et al., 2003	Evaluation of the influence of stiffness (similar tonative ^1^ACL, ^2^BPTB of 10 and 14 mm) and initial tension of grafts upon knee kinematics and biomechanics.	A validated intact knee ^3^FE model (Li et al., 1999) was used whose internal-external moments had been tested against the experimental results from a robotic testing system utilizing a cadaver’s knee.
Au et al., 2005	FE stress analysis of bone tunnels and cortical bone subject to button-type fixation in reference to two different age groups.	The FE model of tibia was validated against *in vitro* strain gage data from Reilly et al. (1982) and Bourne et al. (1984).
Peña et al., 2005	Investigation of the impact of graft stiffness (BPTB, gracilis, and quadrupled semitendinosus graft) and initial tension on the knee biomechanics.	The numerical results regarding anterior tibial translation were compared with those of Suggs et al. (2003).
Peña et al., 2006	Assessment of the angle effect of the tibial and femoral tunnels in ^4^ACLR with BPTB on the knee joint kinematics and meniscal stresses with a 134 N anterior load under different flexion angles.	The existing FE model (Peña et al., 2005) was also tested against the *in vitro* findings of Simmons et al. (2003) concerning graft tension at different flexion angles.
Ramaniraka et al., 2007	Various reconstructions were simulated and evaluated (a healthy knee, intra-articular with single- and double-bundles, extra-articular alone and extra-articular combined with intra-articular) regarding knee kinematics.	The numerically obtained knee kinematics were compared with the clinical results of Skyhar et al. (1993) and Harner et al. (2001).
Chizari et al., 2008	Experimental and numerical investigation of the stress pattern in the tibial tunnels owing to screw fixation.	The porcine tibia’s responding to a bovine tendon loading along the line of the tibial tunnel was tested against authors’ experimental results.
Chizari and Wang, 2009	The methodology concerning the development of a subject-specific FE ACL reconstructed knee model was described step by step along with some preliminary results.	This conference paper did not mention any validation information except for some preliminary results for their paper that follows hereupon.
Chizari et al., 2009	E valuation of the stresses on the tendon and tibia and their relation to healing modes of animal studies with a 200 N tensile load applied to the graft.	The porcine tibia’s respond to a bovine tendon pure tensile load of 200 N along the line of the tibial tunnel was tested against authors’ experimental results.
Chizari et al., 2011	Simulation of the behavior of an ACL reconstructed knee with an interference screw fixation in both single-cycle and cycle loading tests and comparison with experiments by using porcine tibia and bovine tendon.	The above FE model was used by additionally adopting a Moonley–Rivlin hyperelastic model, the coefficients of which were experimentally found.
Kim et al., 2011	Estimation of variation in the length and tension of the double bundles at various flexion angles.	The numerical change of the bundles’ length under different flexion angles was compared with *in vivo* results of Yoo et al. (2010).
Wan et al., 2011	Quantitative analysis of the influence of the effect of different ACL graft reconstructions on the biomechanics of the knee joint.	The numerical results regarding anterior tibial translation were compared with those of Suggs et al. (2003) and Peña et al. (2005).
Abdullah et al., 2012	Investigation of the effect of interference screw material in reference to its stability in ACLR.	The authors did not mention any model verification process. They only referred to the use of a commercial FEA software.
Yao et al., 2012	FE analysis related to the influence of tunnel formation on articular stress deterioration following single- or double-bundle ACLR subject to various loading conditions.	(a) The deformation of the menisci was compared with obtained MR images and (b) the contact areas on tibia under compressive forces were compared with the experimental findings of Fukubayashi and Kurosawa (1980).
Huang et al., 2012	Different ACLR techniques were investigated, including single-bundle, double-tibial single-femoral, double-tibial double-femoral and single-tibial double-femoral under valgus moment of 10 Nm and internal torque of 5 Nm.	The numerical results regarding anterior tibial translation were compared with those of Yagi et al. (2002); Suggs et al. (2003), and Peña et al. (2005).
Westermann et al., 2013	Evaluation of the effect of graft size for the Lachman test, estimation of knee laxity, meniscal stresses and peak contact pressures on articular cartilage.	Comparison of the simulated tibial translation under anterior tibial load with some numerical and experimental studies (Yagi et al., 2002; Brophy and Pearle, 2009; Kato et al., 2013).
Wang et al., 2015	Simulation of the anatomic and transtibial single-bundle ACLR with 2 graft fixation angles under intact, ACL deficient and reconstructed context subject to anterior and quadriceps loads equal to 134 and 400 N, respectively, at various flexion angles.	This study used the validated FE model developed in Suggs et al. (2003).
Bae et al., 2016	Evaluation of the tunnel position and shape, the bending angles of graft and the biomechanical effects on the grafts utilizing either an anatomic transtibial or anteromedial portal technique.	The calculated peak stresses of the tibia-bundle contact were compared with those of Kim et al. (2011).
Halonen et al., 2016	Study of optimal graft prestrain and different ACLR techniques (single- and double-bundle ACLR) during gait.	Authors used previous validated healthy knee FE models (compared against experiments) by replacing ACL with a graft under different ACLR techniques (Halonen et al., 2014).
Vairis et al., 2016	Investigation of the mechanical knee joint behavior by calculating stresses and displacements of an intact ACL, a deficient ACL, and a reconstructed ACL FE model.	Same load scenarios with Bendjaballah et al. (1995) and Moglo and Shirazi-Adl (2003) were performed regarding intact ACL and ACL-deficient knee at 0° of flexion angle.
Westermann et al., 2017	Investigation of knee biomechanics and kinematics for optimizing graft placement close to the anatomic femoral footprint.	This study used the validated FE model developed in Westermann et al. (2013)
Kang and Bae, 2017	Determination of the optimal tunnel starting position on femur under an outside-in surgery technique via stress analysis on the graft, estimation of the graft bending angles, tunnel length by considering continuous motion of the knee.	The authors did not mention any model verification process. They only referred to the use of a commercial FEA software.
Wan et al., 2017	Effect of different lengths of hamstring tendon in ACLR under two common clinical loads, namely an anterior tibial drawer as well as pivot shift.	This study used the validated FE model developed in Wan et al. (2011).
Bae and Cho, 2019	Study of the influence of pretension on the graft in ACLR surgery via stress analysis along with assessment of optimal tunnel position in femur.	Comparison of the calculated graft stresses with the experimental values of Noyes and Grood (1976).
Completo et al., 2019	^4^FE analysis of the effect of the bone tunnels sites on kinetics and stresses distribution and functional outcomes after ACLR.	The kinematics and kinetics from the intact ACL model were in the same range with *in vivo* studies such as Liu et al. (2010).
Tampere et al., 2019	Reaction forces and moments within the graft were calculated for anteromedial portal and transtibial techniques. Moreover, the location of the tunnel concerning the anatomical center of the insertion sites was assessed.	Statistical comparison with the CT imaging study of Clockaerts et al. (2016)
Naghibi et al., 2020	FE analysis, founded on cadaveric experiments, regarding 3 ordinary single-bundle and 1 double-bundle ACLR to determine the optimal graft positions along with graft type and tensioning to restore the kinematics of the knee. To this end, a gait cycle was simulated.	The translational and rotational kinematics of the knee joint under different flexion angles and loading scenarios were tested with the experimental findings of Naghibi Beidokhti et al. (2017)

*^1^ACL, anterior cruciate ligament; ^2^BPTB, bone-patellar tendon-bone; ^3^FE, finite element; ^4^ACLR, anterior cruciate ligament reconstruction.*

## Finite Element Modeling of ACL Reconstruction

### Knee Joint Anatomy

In this subsection, the anatomy of the knee joint is briefly presented in order to obtain a good understanding of the knee’s structural components that need to be considered in the modeling process.

**FIGURE 2:**
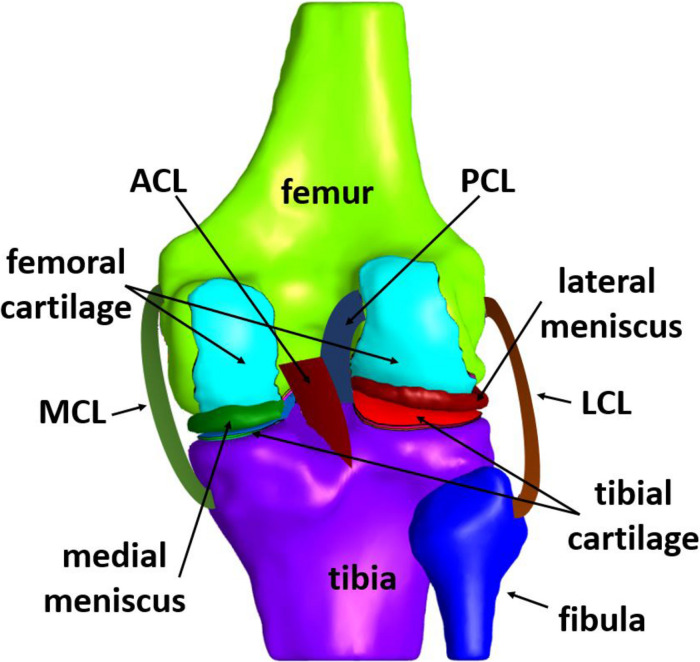
Schematic illustration of the knee joint anatomy.

The knee joint consists of four bones, namely femur, tibia, patella, and fibula. In particular, fibula is situated at the lateral side of the knee, having a similar length to tibia but being much thinner. Each end of the bones has an articular cartilage, which is an avascular sponge-like tissue, enabling the bones to slide along each other with minimal friction (Saarakkala et al., 2010). In addition to cartilages, protection of bones is assured by two menisci, which are crescent-shaped cartilaginous tissues, located between the tibial plateau and femoral condyle (Makris et al., 2011). As a synovial joint itself, a capsule encloses the joint. More specifically, the synovial fluid fills the gap between the cartilages, thus, providing lubrication for the purpose of reducing wear and friction (Schmidt and Sah, 2007). There are four primary ligaments, namely medial and lateral collateral ligaments (usually abbreviated as MCL and LCL, respectively), anterior and posterior cruciate ligaments (usually abbreviated as ACL and PCL, respectively). Moreover, the patellar ligament stabilizes the knee via resisting forces and moments (Claes et al., 2013). The schematic illustration of the tibiofemoral joint anatomy, depicted in Figure 2, includes the bones, their cartilages, both menisci and the four primary ligaments mentioned above, which are typically included in a knee FE model.

### Geometry Modeling and Mesh Generation

The procedure of developing anatomical FE models begins with the acquisition of medical images [MR images or computed tomography (CT) images]. These images are carefully processed in order to obtain a 3D subject-specific representation of the underlying anatomical geometry (Kazemi et al., 2013). Within that process, (1) the medical images are first segmented to describe the boundaries of the anatomical structures, (2) the 3D surfaces of these structures are calculated directly from the 3D reconstruction of the segmented images, and (3) volume meshes are created by filling with FE the volume enclosed by each surface. Additionally, a procedure of smoothing the surfaces can be followed so as to be able to obtain FE meshes with minimum artifacts (e.g., sharp edges) and, as a result, to improve the accuracy of the FE analysis.

Interestingly, MR images are frequently chosen for soft tissue reconstruction while CT images for bones (Kazemi et al., 2013). Also, subject-specific geometrical models may result either by segmenting cadaveric or living subjects. Depending on the research questions to be answered, two-dimensional (2D) representation of the knee joint may also be used in a FE analysis (Federico et al., 2004; Wilson et al., 2005).

**FIGURE 3:**
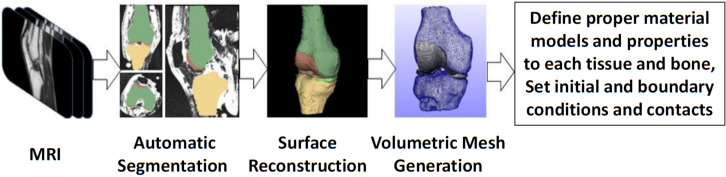
Overview of the modeling and simulation approach for creating subject-specific FE models that are used for detailed analysis of complex movements.

As an illustrative example regarding the development of subject-specific FE knee models, the pipeline used by our group (Nikolopoulos et al., 2020; Stanev et al., 2020) is depicted in Figure 3. In short, the process starts with the collection of the knee joint MR images. Then, the 2D slices are segmented automatically, since automatic methods are scalable to large data sets and reduce the tedious work required by manual methods. The automatic segmentation method may produce geometries that have rough surfaces and irregular components. Hence, these components can be removed and the refined geometries can be used to create volumetric meshes without any significant loss of the anatomical geometry. Volumetric locking in finite elements has been a major concern when modeling the response of incompressible (or almost incompressible) materials such as cartilage. To avoid volumetric locking, hexahedral meshes are generally preferred. However, automatic hexahedral mesh generation is still considered to be a challenging research topic.

**FIGURE 4:**
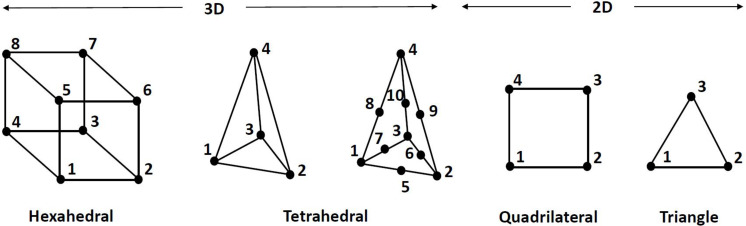
Type of elements used in the reviewed papers.

As can be seen in Table 2 and illustrated in Figure 4, for 3D FE representation, two types of elements are commonly used for the generation of the mesh, namely hexahedral and tetrahedral elements. The former refer to eight-node trilinear hexahedral elements (one node for each corner) that are also called brick elements. Their shape functions in the form of isoparametric coordinates (*r*, *s*, *t*) read:

(1) $$\begin{array}{c} \Psi_{1}=1/8\left(1-r\right)\left(1-s\right)\left(1-t\right),\Psi_{2}=1/8\left(1+r\right)\left(1-s\right)\left(1-t\right),\\ \Psi_{3}=1/8\left(1+r\right)\left(1+s\right)\left(1-t\right),\Psi_{4}=1/8\left(1-r\right)\left(1+s\right)\left(1-t\right),\\ \Psi_{5}=1/8\left(1-r\right)\left(1-s\right)\left(1+t\right),\Psi_{6}=1/8\left(1+r\right)\left(1-s\right)\left(1+t\right),\\ \Psi_{7}=1/8\left(1+r\right)\left(1+s\right)\left(1+t\right),\Psi_{8}=1/8\left(1-r\right)\left(1+s\right)\left(1+t\right). \end{array}$$

**TABLE 2:** Type of joint, surgery treatment, material models, and elements used by the reviewed papers along with the number of elements (whenever these were available).

References	Joint	Bundles	Cartilage	Meniscus	Ligaments/Graft	Bones/other	Total ^1^elements number
Suggs et al., 2003	^2^PF, ^3^TF	^4^SB	^5^LE ^6^iso; 3 layers of ^7^8NBE	3 compression ^8^spr	Nonlinear tensile spr; ^9^ACL: 4, ^10^PCL: 4, ^11^MCL: 5, ^12^LCL: 3, ^13^BPTB: 1	Rigid; ^14^4NSE	^15^N/A
Au et al., 2005	–	SB	Not included	Not included	Not included	Tibia, femur (cortical, cancellous, subchondral): LE iso; ^16^10NTE	376,072
Peña et al., 2005	TF	SB	Not included	Not included	Nonlinear ^17^HE fibered; 8NBE / BPTB	Tibia, femur: rigid / plugs: LE iso; 4,783 4NSE	N/A
Peña et al., 2006	TF	SB	LE iso	LE iso	Nonlinear HE fibered; 8NBE / BPTB	Tibia, femur: rigid / plugs: LE iso; 4NSE	6,909
Ramaniraka et al., 2007	TF	SB, DB	LE iso; 8NBE	LE iso; 8NBE	Nonlinear HE; 8NBE	Rigid; 4NSE	36,500
Chizari et al., 2008	–	SB	Not included	Not included	Only bovine tendon: LE iso; ^18^4NLTE	Tibia (cortical, cancellous): LE iso; 4NSE / screw: LE iso; ^19^4NBQE	N/A
Chizari and Wang, 2009	–	SB	Not included	Not included	Only tendon: LE iso; 2179 4NLTE	Tibia: LE iso; 41,516 4NLTE	43,695
Chizari et al., 2009	–	SB	Not included	Not included	Only bovine tendon: LE iso; 2,179 4NLTE	Porcine tibia: LE iso; 41,516 4NLTE	43,695
Chizari et al., 2011	–	SB	Not included	Not included	Only bovine tendon: ^20^M-R iso; 2,179 4NLTE	Porcine tibia: M-R iso; 41,516 4NLTE	43,695
Kim et al., 2011	TF	DB	Not included	Not included	Only graft (material not mentioned): HE iso	Rigid	N/A
Wan et al., 2011	TF	SB	LE iso	LE ^21^transv iso	BPTB, ^22^DS, ^23^QS: N-H; 4NLTE	Rigid; 4NSE	N/A
Abdullah et al., 2012	TF	SB	Not included	Not included	Not included	Only tibia: LE iso; 4NSE / screw: LE	N/A
Yao et al., 2012	TF	SB, DB	LE iso;	LE transv iso	Nonlinear tensile spr / graft (N/A)	Subchondral, cortical, cancellous: LE iso	N/A
Huang et al., 2012	TF	SB, DB, ^24^SF-DT, ^25^DF-ST	LE iso; 8NBE	LE iso; 8NBE	Nonlinear HE fibered; 8NBE / BPTB	Rigid / plugs: LE iso	N/A
Westermann et al., 2013	TF	SB	^26^HGO; 3 layers of 8NBE	HGO; 8NBE / BPTB	HGO; 8NBE / graft (N/A)	Tibia, femur: rigid; 4NBQE	N/A
Wang et al., 2015	PF, TF	SB	LE iso	Compression springs	Nonlinear tensile spr; ACL: 4, PCL: 4, MCL: 5, LCL: 3, BPTB: 1	Rigid; 4NSE	N/A
Bae et al., 2016	TF	SB	Not included	Not included	Only graft: nonlinear HE fibered; 8NBE / QS	Rigid; ^27^8NQSE	N/A
Halonen et al., 2016	PF, TF	SB, DB	^28^FRPVE; 4 layers of ^29^8NBTDP	^30^FRPE; 4 layers of 8NBTDP	Nonlinear tensile spr / GS	Rigid	N/A
Vairis et al., 2016	TF	SB	LE and ^31^N-H iso	LE and N-H iso	LE and N-H iso / patellar tendon	LE and N-H iso	12,255 (^32^ACLR scenario)
Westermann et al., 2017	TF	SB	HGO; 3 layers of 8NBE	HGO; 8NBE / BPTB	HGO; 8NBE / graft (N/A)	Tibia, femur: rigid; 4NBQE	N/A
Kang and Bae, 2017	TF	SB	Not included	Not included	Only graft (model and type N/A)	Rigid	N/A
Wan et al., 2017	TF	SB	LE iso; 10NTE	LE transv iso; 10NTE	Nonlinear HE fibered; 10NTE / hamstring tendon	Cortical, cancellous: LE iso; 10NTE	N/A
Bae and Cho, 2019	TF	SB	Not included	Not included	Only graft (Model and type N/A)	Rigid	N/A
Completo et al., 2019	TF	SB	LE iso; 10NTE	LE iso; 4NLTE	LE iso; 4NLTE / BPTB	Femur, tibia, fibula: Rigid; ^33^3NTS	100,600
Tampere et al., 2019	TF	SB	Not included	Not included	LE ^34^ortho, 8NBE	Rigid	N/A
Naghibi et al., 2020	PF, TF	SB, DB	Material modeling (N/A); 10NTE	Material modeling (N/A); 10NTE	Nonlinear tensile spr / BPTB, QS, GS	Rigid	N/A

*^1^elem: elements; ^2^PF, patellofemoral; ^3^TF, tibiofemoral; ^4^SB, single-double; ^5^LE, linear elastic; ^6^iso, isotropic; ^7^8NBE, 8-node brick elements; ^8^spr, springs; ^9^ACL, anterior cruciate ligament; ^10^PCL, posterior cruciate ligament; ^11^MCL, medial cruciate collateral ligament; ^12^LCL, lateral cruciate collateral ligament; ^13^BPTB, bone-patellar tendon-bone; ^14^4NSE, 4-node surface elements; ^15^N/A, not available; ^16^10NTE, 10-node tetrahedral elements; ^17^HE, hyperelastic; ^18^4NLTE, 4-node linear tetrahedral elements; ^19^4NBQE, 4-node bilinear quadrilateral elements; ^20^M-R: Mooney–Rivlin HE model; ^21^Transv iso, transversely; ^22^DS, double semitendinosus; ^23^QS, quadruple semitendinosus; ^24^SF-DT, single-femoral double-tibial; ^25^DF-ST, double-femoral single-tibial; ^26^HGO, Holzapfel, Gasser and Ogden HE model; ^27^8NQSE, 8-node quadratic surface element; ^28^FRPVE, fibril-reinforced poroviscoelastic; ^29^8NBETDP, 8-node brick with trilinear displacement and trilinear pore pressure; ^30^FRPE, fibril-reinforced poroelastic; ^31^N-H: Neo-Hookean HE model; ^32^ACLR, anterior cruciate ligament reconstruction; ^33^3NTS, 3-node triangular shell; ^34^ortho, orthotropic.*

The tetrahedral elements were presented in the form of either a linear four-node or ten-node quadratic tetrahedral element. The shape functions of the four-node elements (one node for each corner) are:

(2) $$\Psi_{1}=1-r-s-t,\Psi_{2}=r,\Psi_{3}=s,\Psi_{4}=t$$

while the shape functions of the ten-node elements (four nodes at the corners and six nodes at the edges midpoint) are:

(3) $$\begin{array}{c} \Psi_{1}=t_{i}\left(2t_{i}-1\right),i=1\ldots 4,\\ \Psi_{5}=4t_{1}t_{2},\Psi_{6}=4t_{2}t_{3},\Psi_{7}=4t_{3}t_{1},\\ \Psi_{8}=4t_{1}t_{4},\Psi_{9}=4t_{2}t_{4},\Psi_{10}=4t_{3}t_{4}\\ \text{with}t_{1}=1-r-s-t,t_{2}=r,t_{3}=s,t_{4}=t. \end{array}$$

It should be stressed that simpler representation of the ligaments and grafts via springs was presented, as it will be discussed in section “Material Models and Properties.” Furthermore, when a rigid representation of the bones were used, the discretization was made with shell elements. The four-node quadrilateral shells, shown in Figure 4, are given by the following shape functions in the form of isoparametric coordinates:

(4) $$\begin{array}{c} \Psi_{1}=1/4\left(1-r\right)\left(1-s\right),\Psi_{2}=1/4\left(1+r\right)\left(1-s\right),\\ \Psi_{3}=1/4\left(1+r\right)\left(1+s\right),\Psi_{4}=1/4\left(1-r\right)\left(1+s\right) \end{array}$$

Regarding the three-node shell elements, the shape functions read:

(5) $$\Psi_{1}=1-r-s,\Psi_{2}=r,\Psi_{3}=s$$

Comprehensive description of all types of elements used in FE methodology in structural mechanics can be found in several references, including the theory manual of the open source software FEBio (Maas and Weiss, 2007) from which the above relationships were derived.

As concerns the process of producing the tunnels for the grafts, it can be accomplished via Boolean operations with twisted cylinders so as to closely mimic the surgical intervention. As an indicative methodology, the anatomic transtibial technique implemented by Bae et al. (2016) is briefly analyzed here, where a single-bundle methodology was adopted. Firstly, the femur was rotated from 30° of flexion to 75° and then a 4 mm anterior translation was imposed. Subsequently, a femoral and tibial tunnel drilling of 7.5 mm diameter took place via Boolean operations. The tunnel at the tibia was drilled at 30.4 and 37.0° in the anterior and the lateral view, respectively. Finally, a graft with a diameter of 7 mm was inserted into the tunnels of each bone to connect the two tunnels by minimum distance.

### Loading and Boundary Conditions

The loading and boundary conditions imposed in the FE studies, which were selected for this review, can be partitioned into three main categories.

**FIGURE 5:**
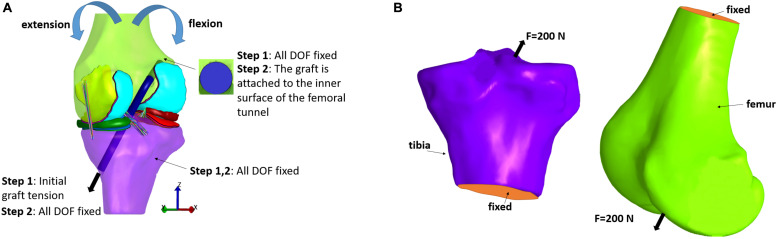
Typical loading and boundary conditions applied in FE modeling of ACL reconstruction according to: **(A)** the first and **(B)** second type of loading scenarios.

In the first category, an anterior load or an internal torque at the tibia is used for evaluating their effect on knee joint kinematics and stresses within the graft. A 3D representation of the graft should be implemented, since a spring representation of it is not suitable for stress distribution calculation. The initial FE modeling set-up is illustrated in Figure 5A. In particular, two steps are usually used to simulate ACLR (see for example, Bae et al., 2016). In the first step, the end graft area of the femur is fixed, while, regarding the corresponding graft area of the tibia, an initial tension is applied, which is aligned with the outward-facing unit normal vector of the graft cross section. Moreover, both the tibia and femur are completely fixed so as to prevent their rotation and translation. In the second step, the tibia is fixed similar to step 1. In the femoral tunnel, the end area of the graft is attached to its inner surface, while the corresponding surface in the tibial tunnel is fixed at its existing location (Bae et al., 2016). Subsequently, femur rotation and translation, forces and moments, can be applied similar to ordinary FE knee simulations according to the studied application. Concerning the present selected studies, a 134 N anterior load was applied to the tibia in Suggs et al. (2003), Peña et al. (2005, 2006), Wan et al. (2011, 2017), Wang et al. (2015). A posterior force of 100 N was applied to the femur in different flexion angles in Naghibi et al. (2020), while ten load cases, from 10 to 100 N, was tested in (Vairis et al., 2016) and an internal tibial torque of 2 Nm was applied in Ramaniraka et al. (2007). In Huang et al. (2012), a combined valgus moment (10 Nm) and internal torque (5 Nm) was simulated. Additionally, the Lachman test was simulated in Westermann et al. (2013, 2017), Tampere et al. (2019), while Kim et al. (2011), Bae et al. (2016), Westermann et al. (2017) used no forces or torques, but only different flexion angles. Finally, in Yao et al. (2012) three different loading scenarios were investigated, namely a compressive load of 1,500 N, a moment of internal rotation and an abducting moment corresponding to the maximum values of a normal walking cycle.

The second category, on the other hand, consists of the investigations which deal with the examination of the fixation devices outcomes on stress distribution at the bones (Au et al., 2005; Chizari et al., 2008, 2009; Chizari and Wang, 2009; Abdullah et al., 2012). In order to estimate stresses caused by compression from graft anchors, such as buttons and screws, the anchor is loaded with a 200 N force which is directed in the direction of the tunnel, as it can be seen in Figure 5B. This loading scenario is used to simulate the graft tension during gait at full extension (Harrington, 1976). Also, the lower tibial or the upper femoral part is considered to be fixed. Fixation between the screw and bone tunnel, for example, was supposed to be completely bonded, while no sliding and separation was permitted between edges and faces in Abdullah et al. (2012).

Finally, the forces simulating a full gait cycle or the stance phase along with flexion angles and internal/external torque were also used as loading and boundary conditions in Naghibi et al. (2020) and Halonen et al. (2016), respectively. Subsequently, the kinematics of all ACL reconstructed cases were compared with the ACL intact and raptured cases.

### Material Modeling and Properties of Grafts

Additionally to the demanding geometrical representation of the knee structures, suitable material models that describe with accuracy the mechanical behavior of each tissue have to be determined. Moreover, proper material properties have to be assigned. When selecting such material models, important structural aspects of the tissue and microstructural arrangements of its constituents are usually considered. The main distinction between the modeling of healthy knee joint FE models and those which focus on ACLR is principally the use of a graft instead of the native ACL. This implies that one should use the material properties of a specific graft instead of the properties of the native ACL. Furthermore, the graft passes through specific tunnels constructed in the tibia and femur according to each surgery methodology.

In this study, we focus on ACLR. Thus, a brief presentation of the material models and properties used in the literature to describe the mechanical behavior of the most utilized grafts is provided below. A discussion of their structural and functional characteristics is presented, since it is the structure that is going to determine the realistic material properties. For a detailed description of the material models used for the rest components of the knee joint including menisci, cartilages, and bones, the reader can refer to review studies such as Kazemi et al. (2013).

In summary, in the majority of the numerical studies, bones were considered to be rigid bodies, as a result of their large values of density and Young’s modulus compared to soft tissues. Nevertheless, if stresses on bones due to fixation devices or grafts were to be calculated, the bone was simulated as linear elastic and was usually divided into cortical and cancellous parts for more realistic representation. In fact, the hard exterior layer of bones constitutes the cortical bone that is much denser than the cancellous bone (also known as spongy or trabecular bone). According to the present literature survey, the Young’s modulus (*E*) and Poisson’s ratio (*v*) of cortical bone were usually around 13.4 GPa and 0.24, respectively, with (Wan et al., 2017) using higher values for them, namely *E* = 16.2 GPa and *v* = 0.36. Regarding the cancellous bone, *E* ranged between 283 and 389 MPa and *v* was approximately 0.3. Less frequently, also subchondral bones were used (Au et al., 2005; Yao et al., 2012) with *E* = 1.15 GPa and *v* = 0.25. In three studies (Peña et al., 2005, 2006; Huang et al., 2012), bone plugs were also utilized and modeled as linear elastic materials with *E* = 14.22 GPa and *v* = 0.3.

Additionally, modeling menisci and cartilages as poroelastic materials, i.e., consisting of an interstitial fluid phase and a solid matrix, seems to be the most reasonable approach, however, a time consuming one. Therefore, usually linear elastic models are used instead of poroelastic models. Concerning the linear elastic representation of cartilages, *E* was usually 5 or 15 MPa and *v* around 0.45. Correspondingly, for menisci *E* was usually 59 MPa and *v* equal to 0.49. When a linear elastic transversely isotropic model was adopted, *E* was equal to 20 MPa in the axial and radial directions and in the circumferential direction equal to 140 MPa. Moreover, *v* was 0.2 in the plane of isotropy and the out-of-plane *v* was equal to 0.3.

The material models used in the reviewed articles of this study are summarized in Table 2 along with the type and number of elements (when these were available). It should be mentioned that 42% of the selected studies did not include menisci and cartilages. In Table 2, also the type of investigated joint is presented (i.e., tibiofemoral or/and patellofemoral) as well as the number of the bundles that were taken into account (single-bundle or/and double-bundle).

Finally, a more realistic representation of the knee’s anatomy should include also the synovial fluid, muscles and tendons. Nevertheless, the above components are usually excluded from the FE models for the sake of simplicity. Especially, the inclusion of the synovial fluid in FE models would require a special time-consuming procedure to include fluid-structure interaction (FSI) during the simulation that couples the laws of fluid dynamics and structural mechanics (Hron and Mádlík, 2007).

#### Structure

Tendons are structurally very similar to ligaments and this is the main reason why they are used as grafts in ACLR. Like other biological soft tissues, ligaments, and tendons are constructed from a ground substance matrix which is reinforced with elastin and collagen, while water constitutes at about two-thirds of the ligament weight (Weiss and Gardiner, 2001). Due to the large content in water, the mechanical behavior of these tissues is usually assumed to be incompressible. Glycolipids, proteoglycans, fibroblasts, and water constitute the matrix, namely the connective tissue that encloses collagen. In particular, proteoglycans, which are the main components of the connective tissue, have a crucial function. Some of them are combined with hyaluronic acid to create molecules that are hydrophilic. Subsequently, the hydrophilic molecules and water make extracellular matrix in a gel-like form, which is associated with the large amount of water in ligaments. The interaction of ground matrix and collagen with water is in charge of the observed behavior of time-dependent viscoelasticity (Weiss and Gardiner, 2001).

**FIGURE 6:**
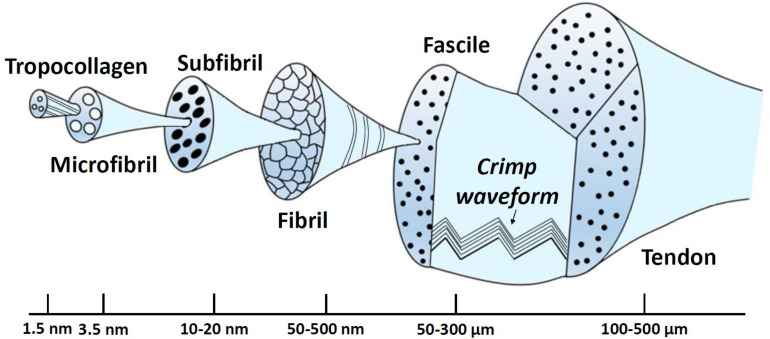
Schematic illustration of the structural hierarchy of tendons.

Collagen molecules follow a structural hierarchy, with the smaller elements packing together to form larger elements which, in turn, are enclosed in even larger structures. The schematic of this structural hierarchy, based on (Kastelic et al., 1978) depiction, is illustrated in Figure 6. In brief, the largest arrangement is the ligament or the tendon, which is divided into smaller components, namely the fascicles. Fascicles enclose the fibroblasts and fibrils. The fibroblasts are cells that take charge of creating the extracellular matrix, while fibrils are assembled together in parallel patterns of fibers. At this level, the crimp (waviness) of the fibril is an instrumental parameter regarding the biomechanics of the tissue. Its biomechanical role is associated with the tissue loading state with increasing loading leading to uncrimping, thus, allowing it to elongate without damage (Frank, 2004). In addition, a fibril is composed of several subfibrils and microfibrils, which in turn, consist of polypeptide chains that have been coiled together to create a tropocollagen molecule (Weiss and Gardiner, 2001). Finally, elastin constitutes a very small percentage of the dry weight of ligaments. Nonetheless, it contributes to the elastic recoverability and the tensile resistance of it (Minns et al., 1973).

#### Material Models and Properties

A key step in modeling ACLR is the selection of the suitable material models and properties for the graft that is going to replace the original ACL. ACL as well as the other ligaments, such as PCL, MCL, and LCL, attaches the bones with each other and provides stability to the knee joint. Various modeling techniques, material models and properties have been adopted in the literature to model knee ligaments (Galbusera et al., 2014). According to the selected papers relevant to ACLR (summarized in Table 1), the constitutive models and material properties for the native ACL and grafts are concisely presented below.

The simplest approach to model the ligaments or grafts is via spring elements. Indicative examples related to the ACLR are the studies of Suggs et al. (2003), Wang et al. (2015). In particular, in these works ACL/grafts were modeled with four nonlinear tensile springs. The following force-strain relationship was used to model their behavior:

(6) $$f=\left\{ \begin{array}{c} 0,\varepsilon <0\\ 0.25k\varepsilon^{2}/\varepsilon_{l},0\leq \varepsilon \leq 2\varepsilon_{l}\\ k\left(\varepsilon -\varepsilon_{l}\right),\varepsilon >2\varepsilon_{l} \end{array}\right.$$

Where ε = (*l*−*l*_0_)/*l*_0_, denotes the strain of the ligament, *l* represents the deformed length and *l*_0_ the length at which the tissue begins to bear the tensile load (Blankevoort and Huiskes, 1991). The constant *k* (the product of Young’s modulus with the cross-sectional area) corresponds to the axial modulus of the linear region of the force-strain relationship and 2ε*_l_* is a critical strain above which the behavior becomes linear. The values of the axial moduli of the ACL and various grafts, which were used by Suggs et al. (2003), along with the corresponding references are summarized in Table 3. Yao et al. (2012) also incorporated the same model for the ligaments, while in Halonen et al. (2016), the gracilis/semitendinosus graft was modeled with nonlinear springs having a stiffness of *k* = 715 N/mm.

**TABLE 3:** Axial modulus (kN) of the ACL and various grafts.

Tissue	Hamner et al., 1999	Frank Noyes et al., 1984	Frank Noyes et al., 1984	Cooper et al., 1993	Woo et al., 1997	Butler et al., 1986	Suggs et al., 2003
^1^ACL	–	4.9	–	–	6.5	13.3	10
^2^BPTB, 10 mm	–	–	–	22.8	–	23.0	20
^2^BPTB, 14 mm	–	18.4	31.0	27.8	–	32.2	30
Semitendinosus	6.4	5.0	15.1	–	–	–	
^3^QST/G	23.3	–	–	–	–	–	

*^1^ACL, anterior cruciate ligament; ^2^BPTB, bone-patellar tendon-bone; ^3^QST/G, quadrupled semitendinosus and gracilis graft.*

The studies of Chizari and Wang (2009), Chizari et al. (2009), Vairis et al. (2016), Completo et al. (2019) used a lineal elastic model for both the native ACL and graft because of the small time of articular loading during knee flexion. More specifically, in Chizari and Wang (2009) and Vairis et al. (2016) the isotropic Young’s modulus and Poisson ratio of the ACL and graft were set according to Kutz (2003) and Reeves et al. (2009), respectively. In Vairis et al. (2016) also a neo-Hookean hyperelastic model was used. Furthermore, in Chizari et al. (2009), an isotropic Poisson’s ratio was also assumed for both the bone and tendon, while experimental tests were performed on animal tissues (bovine flexor tendon and porcine tibia) for the purpose of finding the Young’s moduli. Large linear parts were observed in both stress-strain curves. However, Chizari et al. (2011) experimentally determined the coefficients of the Mooney–Rivlin hyperelastic model (only first order constants) for the tendon and tibia. In order to consider also tendon viscoelasticity, the Rayleigh damping model was adopted.

Three different grafts for the ACLR were investigated in Wan et al. (2011) and a neo-Hookean hyperelastic model for ligaments and grafts was used, assuming that the tissues behave isotropically. In particular, the parameters *C*_1_ of the neo-Hookean model for bone-patellar tendon-bone (BPTB) as well as double and quadruple semitendinosus were equal to 58.23, 19.37, and 19.37 MPa, respectively. The average cross-sectional area of the cylindrical shaped grafts was equal to 33.4 mm^2^ for the BPTB graft, whereas the areas were 23.3 and 55 mm^2^ for the double and quadruple semitendinosus grafts, respectively. The shear modulus in the neo-Hookean model for BPTB as well as double and quadruple semitendinosus was assigned as 116.46, 38.74, and 38.74 MPa, respectively.

Tampere et al. (2019) modeled the isotropic ground substance matrix using the neo-Hookean relationship $\left(W=C_{1}\left({\bar{I}}_{1}-3\right)\right)$. Subsequently, several springs were added which span the 3D ligaments between their attachment points. An exponential function of the displacement, *x*, was used for modeling the force response of each spring:

(7) $$f=\alpha \left[e^{\beta \left(x-x_{0}\right)}-1\right]$$

In the above relationship, *x*_0_ was introduced for the purpose of defining a pre-stretch of either the ligament or the tendon. The constant α is associated with the initial stiffness of the spring and β influences its degree of nonlinearity. The following values, namely α = 7.36, β = 4.35, and *x*_0_ = 0 mm, were used for the above parameters based on fitting experimental data using the least squares regression method. Finally, the parameter *C*_1_ (with *C*_1_ = 2*G*) of the neo-Hookean model was set equal to 1.95 MPa and *D* = 1/*K* = 0.00683 MPa^–1^, with *G* and *K* being the shear and bulk modulus, respectively.

In Peña et al. (2005, 2006), Huang et al. (2012), Bae et al. (2016), and Wan et al. (2017), grafts were considered as fiber reinforced materials, while their constitutive behavior was modeled as hyperelastic, transversely isotropic and near-incompressible, following (Weiss et al., 1996), described by the strain energy density function:

(8) $$W=W_{\text{iso}}+W_{\text{aniso}}+W_{\upsilon }$$

where $W_{\text{iso}}=C_{1}\left({\bar{I}}_{1}-3\right)$ is associated with the mechanical contribution of the ground matrix via a neo-Hookean model, *W*_aniso_ is related to the contribution of the collagen fibers and $W_{\upsilon }=\frac{1}{D}{\left[ln\left(J\right)\right]}^{2}$ is the volumetric part which controls the incompressibility of the tissue. In the above equations, ${\bar{I}}_{1}=J^{-2/3}I_{1},I_{1}=trC$ and *C* = *F^T^F*. C is the right Cauchy–Green deformation tensor, J = *detF*, **F** denotes the deformation gradient and *C*_1_ is a model parameter.

For the anisotropic part, the stress-stretch relationship in the direction of the fibers, assuming that fibers support only tension, is given by:

(9) $$\sigma_{f}=\lambda \frac{\partial W_{\text{aniso}}}{\partial \lambda }\left\{ \begin{array}{c} 0,\lambda \leq 1\\ C_{2}\left(e^{C_{3}\left(\lambda -1\right)}-1\right),1<\lambda <\lambda^{*}\\ C_{4}\lambda +C_{5},\lambda \geq \lambda^{*} \end{array}\right.$$

Where $\lambda =\sqrt{m_{0}\cdot C\cdot m_{0}}$ represents the axial stretch ratio in the fiber direction, *m*_0_ corresponds to the unit vector along the direction of fiber in the undeformed configuration, λ^∗^ denotes the stretch at which the behavior becomes linear and *C*_2_, *C*_3_, *C*_4_, *C*_5_ stand for model parameters. Note that *C*_4_, *C*_5_ are defined so that σ*_f_* is *C*_0_ and *C*_1_ is continuous at λ = λ^∗^. The values of the parameters considered in the above studies for the native ACL and the three grafts, namely the gracilis, the quadrupled semitendinosus and the patellar tendon are depicted in Table 4.

**TABLE 4:** Material parameters for the ACL and grafts (MPa) according to Butler et al. (1990), Gardiner and Weiss (2003), Suggs et al. (2003), and Peña et al. (2005).

Tissue	*C*_1_	*C*_2_	*C*_3_	*C*_4_	λ*	*D*
^1^ACL	1.95	0.0139	116.22	535.039	1.046	0.00683
Patellar tendon	2.75	0.065	115.89	777.56	1.042	0.00484
Semitendinosus	2.75	0.065	115.89	512.73	1.042	0.00484
Gracilis	2.75	0.065	115.89	791.4	1.042	0.00484

*^1^ACL: anterior cruciate ligament.*

A non-linear hyperelastic law for the ligaments and patellar tendon graft was used by Ramaniraka et al. (2007), which was developed by Pioletti et al. (1998). More specifically, the strain energy reads as follows:

(10) $$W=a\text{exp}\left[b\left(I_{1}-3\right)-\frac{ab}{2}\left(I_{2}-1\right)\right]$$

with *a* and *b* being material constants and *I*_1_, *I*_2_ strain invariants. The mean values of the material constants were derived from experimental measurements. In particular, *a* = 0.3 MPa, *b* = 12.2 for ACL, while *a* = 0.09 MPa, *b* = 66.96 for the patellar tendon graft.

The HGO model was used by Westermann et al. (2013, 2017) for the representation of the ligaments’ mechanical behavior of the Gasser et al. (2005), which is considered a distribution of the collagen fiber orientations in the tissue. The strain-energy potential for this model has the following form:

(11) $$\begin{array}{c} U=C_{10}\left(I_{1}-3\right)+\frac{1}{D}\left[\frac{{\left(J^{el}\right)}^{2}-1}{2}-lnJ^{el}\right]\\ +\frac{k_{1}}{2k_{2}}\sum_{a=1}^{N}\left\{\text{exp}\left[k_{2}{\left(\kappa \left(I_{1}-3\right)+\left(1-3\kappa \right)\left(I_{4\left(aa\right)}-1\right)\right)}^{2}\right]-1\right\} \end{array}$$

In the above relationship, the number of fiber families within the tissue is represented by *N*, the symbols *k*_1_, *k*_2_, *D*, *C*_10_ correspond to material parameters and *J*^el^ represents the elastic volume ratio. Also, *I*_1_ denotes the first strain invariant and *I*_4(*aa*)_ are the pseudo-invariants equal to the square of the stretch in the preferred fiber orientation of each fiber family. The heterogeneity of the distribution of the fiber directions is quantified by *k*, while the dominant orientation of the fibers was presumed to be parallel to the graft long axis.

### Verification and Validation Assessment

A key point in FE analysis, as with all computational methods, is the comprehension of the difference between three dissimilar but closely related words, namely “model,” “code,” and “simulation.” In effect, one applies a model into a computer code. Afterward, this code is utilized to carry out a simulation which can yield values to be used in the biomechanical analysis. Reliability is acquired by displaying acceptable uncertainty and error levels. These levels are established via verification and validation assessments, which, in plain words, are the areas of mathematics and physics, respectively.

In particular, verification has to do with the procedure that identifies whether the programming and the model’s implementation are correct. It has two main branches, which are code verification (solution algorithms and mathematical model are correct) and calculation verification (accurate discrete solution of mathematics). In FE analysis, some ordinary items to be checked in the verification process are the geometry (Does it agree with the real case?), mesh refinement (Is the mesh adequately refined to give accuracy?), the material properties and model (Are they realistic?), element properties (Are they in line with shape distortion criteria? Do they fill the entire geometry?), applied loads and contact between components (Are they properly applied?) and outputs (Are they continuous across elements and comparable with hand calculations?).

In contrast, validation assessment determines if the model agrees with real world’s representation. It has also two components, namely validation experiments (experiments closely related to the validation of the numerical model at hand) and accuracy assessment (appraising the comparison of the computational and experimental results). Nevertheless, most experiments are not usually carried out for validation purposes. Furthermore, a usual validation approach is the comparison of model’s results with a benchmark test or a similar validated FE model for a relatively easy simulation.

To sum up, both assessments consist the backbone of a proper quality assurance plan related to a FE analysis and are usually referred to as “V&V” procedure (Guide for Verification & Validation in Computational Solid Mechanics - ASME, 2020). Consequently, V&V plan is of major importance, especially in biomechanics, since it is the solid foundation of evaluating the model and defining the criteria for the sake of approving the models as appropriate for making credible predictions associated with the planning, assessment and improvement of clinical treatments.

As far as the present review is concerned, a lack of presentation of mesh-refinement processes was observed in the majority of the studies. Besides, as can be gleaned from Table 2, no presentation of the number of elements of the FE model was noted at approximately 70% of the studies.

The validation method, which was followed by each FE model simulating ACLR, can be seen in the second column of Table 1. In brief, the first, to our knowledge, relative presented FE model was that of Suggs et al. (2003). This study utilized a previous intact knee FE model (Li et al., 1999) that had been validated against the experimental results of the internal-external moments derived from a cadaver knee which was subject to internal-external loading. The difference with Li et al. (1999) was the use of a graft instead of the native ACL. The results of Suggs et al. (2003) pertaining to tibial translation owing to a 134 N anterior tibial load served as a benchmark study for Peña et al. (2005), Wan et al. (2011), and Huang et al. (2012). Moreover, the same FE model was used in Wang et al. (2015). Similar to Suggs et al. (2003) and Halonen et al. (2016) modified their previous validated intact knee model by replacing ACL with a graft.

Some authors carried out their own experiments (Chizari et al., 2008, 2009) as a means to verify their computational model by either utilizing cadaveric or animal specimens or used existing experimental results (both *in vivo* and *in vitro*) (Au et al., 2005; Peña et al., 2006; Kim et al., 2011; Yao et al., 2012; Vairis et al., 2016; Bae and Cho, 2019; Completo et al., 2019; Naghibi et al., 2020). Clinical studies were used for validation by Ramaniraka et al. (2007), while both numerical and experimental results were used by Westermann et al. (2013, 2017). The work of Kim et al. (2011) was used as a validation case study by Bae et al. (2016). Finally, three papers did not refer to any validation assessment (Chizari et al., 2008; Abdullah et al., 2012; Kang and Bae, 2017) presenting preliminary results or mentioned the use of commercial FE analysis softwares.

## Simulation of Different Surgery Techniques

### Single- or Double-Bundle ACL Reconstruction

Realistic simulation of ACLR requires adequate knowledge of the steps followed during the surgical operation and biomechanical function of the native ACL. ACL is typically divided into two bundles, namely the anteromedial and posterolateral one. The aforementioned terms have been selected due to their insertion sites into the tibial plateau. The posterolateral bundle is tight when the knee is extended, whereas the anteromedial one is relaxed. Conversely, during knee flexion the anteromedial bundle is tight, while the posterolateral one is relaxed (Girgis et al., 1975; Fu et al., 1999; Zantop et al., 2006). In fact, during knee extension the anteromedial and posterolateral bundles are aligned to the sagittal plane. During flexion, however, the two bundles form a “twisting” pattern (Buoncristiani et al., 2006).

An ideal ACLR should closely mimic the above functionality of the two bundles. In the double-bundle ACLR, the two bundles act individually imitating the original ACL crossing pattern (Amis and Dawkins, 1991). This cannot be achieved in the case of the single-bundle surgery. As a consequence, the so-called anatomic double-bundle ACLR seems to provide a better biomechanical outcome and restore the knee kinematics to a greater extent (Yasuda et al., 2011).

**FIGURE 7:**
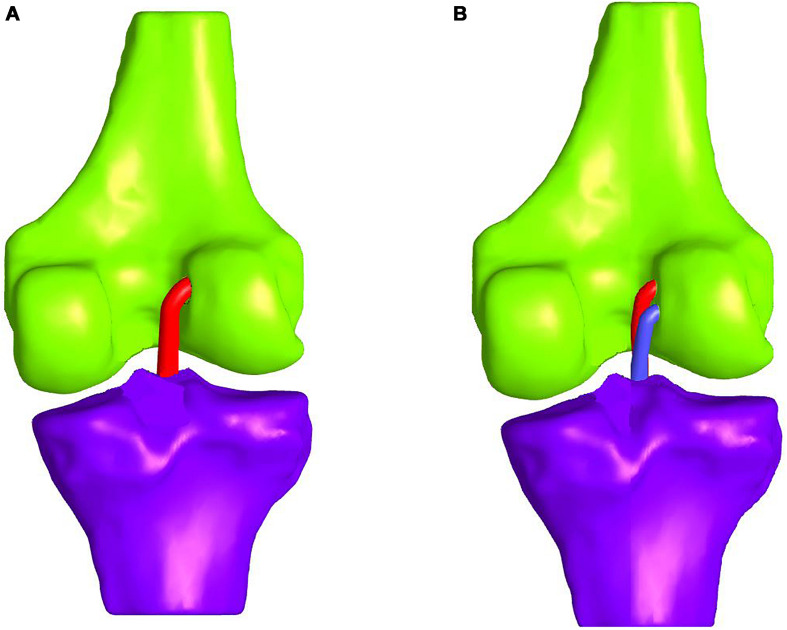
Schematic representation of: **(A)** single-bundle and **(B)** double-bundle ACL reconstruction (only grafts and bones are depicted for the sake of clarity).

In comparative studies, such as Järvelä (2007), the double-bundle ACLR appeared to provide a better rotational stability at a 14-month follow-up evaluation. On the contrary, single-bundle ACLR leads to significantly more graft failure, thus, resulting in more ACL revision surgeries (Suomalainen et al., 2012; Järvelä et al., 2017). The difference in graft failure rates can be attributed to the difference in overall graft thickness between the two methods. In the double-bundle treatment, the anteromedial graft is usually selected to be about 7 mm and the posterolateral around 6 mm thick. Hence, a total diameter of 13 mm arises compared to the single-bundle that is usually 7–8 mm (Järvelä et al., 2017). On the whole, the double-bundle ACLR appears to be more durable, even though there are not enough long-term follow-ups (Järvelä et al., 2017). A schematic representation of the single- and double-bundle ACLR is illustrated in Figures 7A,B, respectively. For the sake of clarity, tissues and patella are not depicted.

Concerning the FE modeling, most of the studies attempted to simulate the single-bundle technique (Chizari and Wang, 2009; Suggs et al., 2003; Peña et al., 2005, 2006; Chizari et al., 2009, 2011; Wan et al., 2011, 2017; Westermann et al., 2013, 2017; Wang et al., 2015; Bae et al., 2016; Vairis et al., 2016; Kang and Bae, 2017; Bae and Cho, 2019; Tampere et al., 2019; Completo et al., 2019). In particular, in Suggs et al. (2003), Wang et al. (2015) a single nonlinear spring was used to capture the behavior of the ACL and the grafts. Consequently, they were not able to examine the stress distribution within the graft/ACL. The utilization of 3D geometries for the grafts enabled to investigate the aforementioned stress distributions. In Chizari and Wang (2009), also the stresses were calculated at the tibial cortical bone within the tibial tunnel by accounting only for the tensile load of the tendon graft.

Unlike the previously mentioned publications, Kim et al. (2011) simulated the double-bundle ACLR at four flexion angles, namely 0, 45, 90, and 135° with the intention of calculating the alteration of the length and tension. The two bundles were inserted into the tunnels under 20 N of initial tension before the fixation. The length of the anteromedial bundle at knee flexion was found to be gradually reduced between 45 and 90° and then retain a constant value. On the other hand, the length of the posterolateral bundle declined at 45 and 90° and then gradually increased at 135°. As far as the reaction force of the two bundles is concerned, that of the anteromedial bundle decreased at 45°, but then it was invariable until full knee flexion. On the contrary, the reaction force of the posterolateral bundle reduced at 45 and 90° and then increased at full flexion.

Finally, four investigations provide a comparison between the single-bundle and double-bundle ACLR, which are the studies of Ramaniraka et al. (2007), Yao et al. (2012), Halonen et al. (2016), and Naghibi et al. (2020). In Ramaniraka et al. (2007), the extra-articular and combined intra- and extra-articular procedures were evaluated with respect to ACLR. The single-bundle intra-articular method aims at substituting the anteromedial bundle, whereas the double-bundle intra-articular technique at substituting both the anteromedial and posterolateral bundles. In contrast, extra-articular procedure utilizes a fascia lata band, distally based on Gerdy’s tubercle, which is slipped under LCL and then threaded closenessly through the femoral tunnel. It was inferred that combined methods can restore the knee stability and its function in most of the ACL deficient knees (Bak et al., 2001). In Ramaniraka et al. (2007), FE models were developed of a healthy knee, intra-articular reconstruction via single- and double-bundle, extra-articular ACLR alone and extra-articular in combination with intra-articular reconstructions. The internal rotation along with the stresses within the ACL and the patellar tendon graft were estimated, which are induced owing to a 2 Nm internal torque. The intra-articular ACLR for single- and double-bundles proved to cause comparable internal rotation and function as the healthy knee joint. Nevertheless, extra-articular ACLR was observed to decrease noticeably the internal rotation and alter the stress distribution in the soft tissues in comparison to the intact ACL.

The study of Yao et al. (2012) demonstrated that the tunneling can induce stress in the articular surfaces. In the case of double tunneling, further deterioration in the stress concentration between the anteromedial and posterolateral tunnels has been observed. Clinically, it is very common for tunnel communication to take place after the surgery, which can be attributed to the high stresses between the anteromedial and posterolateral tunnels (Yao et al., 2012). Generally, bone tunnel enlargement can lead to undesired knee laxity.

In Naghibi et al. (2020), tibial and femoral insertion sites of the graft and its fixation tension were investigated to get similar laxity to intact knee regarding three single-bundle and one double-bundle ACLR. A full gait cycle was used with the native, ACL-ruptured, non-optimal ACL-reconstructed as well as optimal reconstructed knee joints. The results of single-bundle ACLR revealed that for hamstring and patellar tendon grafts anatomical sites (with a fixation force of 40 N) could recover the kinematics and kinetics of the healthy knee. The same can be achieved also with quadriceps tendon with isometric positioning (fixation tension of 85 N). Regarding the double-bundle ACLR, both bundles required a 50 N fixation force at the optimal insertion positions. In a nutshell, all the surgical techniques (i.e., single- and double-bundle ACLR) and graft types can be applied to recover the biomechanical behavior of intact knee. Additionally, Halonen et al. (2016) found that both the single- and double-bundle ACLR can restore the rotational and translational knee motions. However, they observed to increase the strains and stresses on the cartilage.

Interestingly, also additional ACLR techniques were studied, apart from single- and double-bundle, namely double-tibial single-femoral and single-tibial double-femoral tunnel reconstruction. Overall, the double-bundle ACLR proved to be the most advantageous in terms of restoring rotational stability as well as stresses within the grafts and other soft tissues.

### Graft Options

The graft should imitate the anatomy of the real ACL and yield similar biomechanical properties. The optimal graft selection is a combination of surgeon’s experience, graft’s availability, patient’s preference, age, gender, contaminant operations, and activity level (Kim et al., 2013; Vaishya et al., 2015). The grafts can be either biological or synthetic. The former constitute a more popular choice, because they permit easy incorporation into the joint and graft remodeling (Fu et al., 1999). Biological grafts are subdivided into autografts and allografts, which is the standard subdivision based on whether the tissue is obtained from the patient or from a donor. In contrast, synthetic grafts are artificial materials which mimic the behavior of native ACL.

The autografts can be extracted from the BPTB, the four-strand hamstring tendon (semitendinosus and gracilis tendons) and more rarely from the quadriceps tendons (Kim et al., 2013). The BPTB option exhibits high tensile load, high stiffness, better healing owing to bone-to-bone fixation (Fu et al., 1999) and a return to high-level sports activity (Vaishya et al., 2015). Nevertheless, it is related to donor site morbidity. The hamstring tendon, on the other hand, is a reliable graft regarding patients exhibiting low pain tolerance and jobs that include kneeling, while less donor site morbidity has been observed (Dhammi et al., 2015). In addition, the graft harvesting needs a relatively small incision, thus, assuring better cosmetic result. The hamstring tendon, however, is characterized by slower healing process and high incidence of tunnel widening (Kim et al., 2013). Finally, quadriceps tendon harvesting does not violate the fat pad and patellar tendon, thus, preventing the patella baja risk (Beasley et al., 2005). Nevertheless, the long-term data for this graft are scant and it is not recommended for primary ACLR.

As an alternative, for the purpose of eliminating the morbidity that is related to autografts, allografts are gaining popularity (Beasley et al., 2005). According to clinical and animal data, the tissues of allografts revascularize and turn out to be viable after implantation just like autografts (Arnoczky et al., 1986). The allogenic grafts, which are frequently used are the BPTB, hamstring tendon, Achilles as well as anterior and posterior tibialis tendons (Fu et al., 1999; Beasley et al., 2005; Carey et al., 2009; Duquin et al., 2009; Vaishya et al., 2015). Allografts reduce the surgical time and patient’s pain while, at the same time, offer the optimal esthetic outcome by virtue of no incision for graft harvesting. The high risk for disease transmission, the slow graft incorporation, the poor graft strength because of sterilization and increased cost are the main drawbacks (Fu et al., 1999; Kim et al., 2013). Several studies have paid special attention to the comparison of outcomes between autografts and allografts, such as Kustos et al. (2004) and Marrale et al. (2007). As a general remark, all the aforementioned investigations observed no statistically considerable differences in patients’ pain, pivot shift, Lachman testing, and range of motion. Hence, allografts could be a reliable alternative, provided that preservation and sterilization methods do not weaken the tissue and careful screening for viral diseases is accomplished. In a recent cost-effectiveness analysis (Mistry et al., 2019), it was concluded that the cost is the key factor, making autografts the primary preference nowadays. In addition, under certain circumstances, mainly in cases of revision of ACLR, allogenic grafts may be the only option.

The initial enthusiasm for artificial grafts in the 1980s faded away as a consequence of the substantial disadvantages. These drawbacks include the high cost, possible graft fragmentation, cross-infections, and debris dispersion that may result in frequent instability, chronic effusions, synovitis, and osteoarthritis, as highlighted in the review studies of Legnani et al. (2010) and Vaishya et al. (2015). Recently, a resurgence of the enthusiasm has been observed in the investigation of synthetic substitutes such as polypropylene, polyester, Dacron, and carbon fibers. In summary, in spite of many experimental studies being conducted, all materials that have been explored have serious shortcomings. The ideal synthetic graft needs to be biocompatible, have chemical stability and pores for the ingrowth of fibroblasts along with optimal mechanical properties close to the native ACL (Legnani et al., 2010). The reduction of surgery time, the absence of the possibility for donor morbidity and disease transmission may overcome synthetic graft disadvantages and be routinely used in the future, principally in revision surgeries (Cerulli et al., 2013).

In chronological order, Suggs et al. (2003) incorporated a single-bundle reconstruction comparing three graft choices. The first graft had the same axial modulus as the intact ACL, while the second and the third ones were BPTB grafts of 10 and 14 mm. The material properties of the above mentioned grafts are provided in Table 3. As emphasized in Suggs et al. (2003), the graft axial modulus has a significant influence on the knee kinematics. In particular, when initial tension was used, the first graft under-constrained the knee, the second resulted in slight over-constrain and the third in over-constrain comparing to the intact knee. Also, the grafts were inserted at the mid-length of the tunnels and, consequently, the length of the graft is approximately two times larger comparing to that of the intact ACL. Using the same graft modeling and properties for the case of BPTB (10 mm in diameter), Wang et al. (2015) simulated the anatomic and transtibial single-bundle ACLR with two graft fixation angles under intact, ACL deficient and reconstructed context subject to anterior and quadriceps loads equal to 134 and 400 N, respectively, at flexion angles equal to 0, 30, 60, and 90°. With the graft being fixed at 0°, the scenario of the anatomic ACLR led to somewhat higher lateral contact forces at 0° of flexion when compared with the intact knee joint. Regarding the transtibial technique, it resulted in larger contact forces at both 0 and 30° when the same muscle load was implemented. For the case of the graft fixed at 30°, the anatomic ACLR was observed to overstrain the knee at 0° causing larger contact forces, whereas with the transtibial technique being adopted, marginally larger contact forces were noted at 30°.

Three different grafts were compared in Peña et al. (2005), namely the patellar tendon, the gracilis, and the quadrupled semitendinosus in order to investigate the outcome of the graft stiffness on the anterior tibial translation. A comparison was made under the same initial graft tension (40 N) and anterior tibial load (134 N). In short, the anterior tibial translation having a semitendinosus graft led to the highest translation, namely 10.48 mm. No significant dissimilarities between the patellar and gracilis tendon grafts were observed (9.93 and 9.91 mm translation, respectively). Furthermore, the posterior graft femoral insertion displayed the maximal principal stresses, while at full extension, because of the lower stiffness concerning the semitendinosus graft, the lower stress (5.21 MPa) was mentioned. The maximum principal stress was equal to 6.4 and 6.8 MPa for the patellar tendon graft and the gracilis tendon graft, respectively. In Peña et al. (2006), a single graft was used, namely a BPTB, with the same graft pretension and anterior tibial load as Peña et al. (2005), in order to study the influence of the angle in the coronal plane of the tunnels of tibia and femur. Firstly, the tension of the graft was estimated for a 0–60° knee flexion, while the resulting kinematics with a 134 N anterior load was compared with the case of healthy knee. The results revealed that tibial and femoral tunnels with 60° demonstrate the closest results to the native ACL concerning the anterior tibial translation. In this cases, a smaller graft tension was observed. The findings also indicated considerable increases of the stresses on the menisci after ACLR.

Conversely, the study of Ramaniraka et al. (2007) focused on the influence of various ACLR techniques, as it was detailed in the previous subsection, under an internal torque of the tibia equal to 2 Nm at 0, 15, 30, and 45° of flexion. The only graft which was utilized was that of patellar tendon and, thus, no parametric study was conducted to quantify the effect of different graft selection on the ACLR. In the same manner, (Peña et al., 2006; Chizari and Wang, 2009; Kim et al., 2011) used a single graft option.

In the study of Wan et al. (2011), a comparative investigation was conducted to evaluate the effect of three different grafts on the knee joint biomechanics. The utilized grafts were the BPTB, the double as well as the quadruple semitendinosus. A FE model of the knee joint was developed, which encompasses the 3D geometries of menisci, cartilages and the three ligaments, namely MCL, LCL, and ACL/graft. A 134 N force was applied to femur in the posterior direction with the femur being constrained at 0° of flexion. PCL was not included in the numerical model, because of its slack during femur’s posterior translation. Overall, the quadruple semitendinosus graft better restored the knee kinematics as compared to the other grafts. Nevertheless, none of these grafts can potentially restore the ACL function completely. In particular, regarding the case of quadruple semitendinosus, the Mises stresses located in the tibial attachment of MCL were larger than the case of intact ACL. In addition, higher stresses emerge in the posterior area of both lateral and medial menisci. The authors concluded that the higher bearing of the tissues may result in possible damage of MCL and menisci, although this behavior has not been demonstrated in clinical results.

In Naghibi et al. (2020), three grafts were used for single-bundle ACLR, namely BPTB, quadriceps and hamstring tendons, while for the double-bundle model the hamstring tendon was implemented. Their results suggest that all graft types can be used to recover the intact ACL biomechanical behavior. However, regarding the single reconstructive cases, if the placement parameters are not optimized, anatomical sites can lead to better knee laxity restoration.

### Graft Fixation

The fixation of the graft is considered to be vital regarding the long-term outcome of ACLR. The ideal fixation device should provide secure graft fixation, thus, allowing healing within the tunnel and permitting range of motion and weight-bearing exercises (Vaishya et al., 2015). Furthermore, assuring of early return to sports activities without any fixation strength loss turns out to be of major importance (Kim et al., 2013). The strength of fixation is affected by several factors, among which are the bone density and the screw’s geometry and material (Weiler et al., 2000). The fixation devices are generally divided into those used for bone plugs and soft tissues.

On the one hand, bone plugs are commonly fixed with metal and bio-interference screws. The latter have recently become popular and provide similar outcomes with metal screws. Bioabsorbable screws offer no requirement for implant removal, incorporation within the tissue and reduced interference with MR imaging (Kim et al., 2013). Furthermore, cross biodegradable and metal pins are implemented for bone plug fixation. According to Harilainen et al. (2005), interference screws with bone plugs and cross pins exhibit comparable fixation strength.

On the other hand, Retroscrews and interference screws are also used for soft tissue fixation. Moreover, Endobutton has been used for hamstring graft fixation (Beasley et al., 2005). It has less stiffness and a higher load failure than interference screws, while it is adequate for both single- and double-bundle ACLR (Kim et al., 2013). However, Endobutton is responsible for graft motion within the tunnel, thus, potentially causing a widening of the tunnel (Stener et al., 2010). In conclusion, in spite of the disadvantages of the graft fixation devices, the majority of contemporary devices are strong enough to fix the graft in place (Beasley et al., 2005; Vaishya et al., 2015). Hence, the choice of the ideal device should depend on the graft type, experience of the surgeon as well as the patient’s condition.

For the purpose of investigating the stresses at the bone structures via FE analysis, a rigid representation is inappropriate. Instead, the bone is typically partitioned based on anatomical details. Linear elastic models have been used to calculate the stresses and strains at the bones. Cortical bone damage may be observed because of the repetitive compression from the button and possible fracture located at the site of the button fixation (Turner et al., 2001). Au et al. (2005) developed a FE model as a means to examine the cortical stresses on the femur owing to the button-type fixation regarding two age groups. The femur was divided into the cortical, cancellous, and subchondral parts, while linear elastic models were incorporated to describe their mechanical behavior. In addition, two coaxial tunnels were constructed within the bone so as to represent the procedure in the surgery of ACLR. Four points were used for the contact on the button with the cortex and a force of 200 N was applied to the button, which was directed along the axis of the tunnel (estimation for the graft tension during gait at full extension (Harrington, 1976). The simulations demonstrated that cortical stresses, originated from the button fixation, could be high enough (up to 100 MPa at the tunnel aperture) during normal gait and not significantly different for the two age groups, namely 45 and 65 years old.

Chizari et al. (2008) examined both experimentally and numerically the tibial fixation of the screw. This type of fixation is regarded as more problematic comparing to the femoral one, since the forces on the graft are directly parallel with the tunnel (Brand et al., 2000). The bone was divided into two parts, namely the cortical and the cancellous parts. Both components were described as linear elastic materials, while their properties were determined using experimental data and empirical relationships. Furthermore, the Poisson’s ratio was assumed to be isotropic. Apart from a porcine bone, the experimental and numerical studies included a bovine tendon graft and an interface screw. An elastic model was used for the screw that is in contact with the wall of the tibia. The graft was also modeled as an elastic material and the length and diameter of the tibial tunnel were 30 and 9 mm, respectively, with the tunnels drilled at sites defined by Fu et al. (2000). Various stages for fixation were tested in order to evaluate the stresses of the interface screw and the bone. Similar to Au et al. (2005), a 200 N load was applied to the screw along with a rotational movement. The results indicated that the stresses in the tunnel wall were between 10 and 20 MPa. The highest values took place between the sharp screw threads and the tunnel wall, whereas the lowest ones in the tunnel distal end. The main discrepancy between the study of Chizari et al. (2008) and the clinical case is the use of tibia and graft originated from animals. Finally, unlike (Au et al., 2005), this investigation did not consider the impact of age.

Chizari and Wang (2009) derived some preliminary results by applying a pure tensile load on the graft, similar to all FE studies presented in this subsection. The fixation device, namely the bio-absorbable screw, was not included in the numerical model since it was supposed to have been absorbed into the bone. A maximal stress of 0.199 MPa was estimated on the tendon graft at the fixation zone in the tibial tunnel. Finally, the stresses were lower on the cortical bone as compared to those of the cancellous bone. In Chizari et al. (2011), the graft was connected with the tibial tunnel by using a 0.25 friction coefficient representing the interference screw fixation. For a 400 N load in the single-cycle loading scenario, a maximum stress, namely 18.8 MPa, was calculated on the graft at the proximal tunnel end. Regarding the cyclic loading results, the maximum stress at the proximal graft end (following the 1,000 cycle of loading of 400 N), with a 1 Hz frequency was 10.7 MPa.

Finally, Abdullah et al. (2012) investigated the influence of various screw materials in ACLR. The materials that were selected to represent the interference screws were both bio-absorbable materials and metallic biomaterials. The former included poly-lactic acid, poly-lactic co-glycolic acid, and poly-caprolactone, while the latter involved stainless steel and titanium alloy. In a similar manner to the two above studies, a tensile force of 200 N was applied as a first approach to simulate the tension on the graft at full extension throughout gait. The upper surface of the femur and lower one of the tibia were fixed in all directions, as illustrated in Figure 5B. The FE simulations demonstrated that at the distal part of the femur, the highest stresses at the interference screw appeared for stainless steel and the lowest for the poly-lactic acid material. The highest stresses of the other bio-absorbable materials were also lower in comparison to the metallic materials, while titanium alloy screws exhibited lower values than those of stainless steel.

Comparable trends also appeared concerning the screw fixation of the proximal tibia. The highest values developed again in the case of stainless steel, followed by titanium alloy. For the bio-absorbable materials, the highest values (lower than the metallic ones) emerged for poly-lactic acid, followed by poly-lactic co-glycolic acid and poly-caprolactone. Additionally, displacement in reference to the fixation of the interference screw was utilized in order to predict the primary ACLR stability. Briefly, the bio-absorbable screws demonstrated lower displacements comparing to metallic biomaterials. Thus, primary fixation stability was accomplished, which ultimately provided more reliable fixation of the graft. In particular, at both distal femur and proximal tibia regions screws made of poly-lactic co-glycolic acid exhibited the largest displacement and the stainless-steel screws the lowest. In a nutshell, stiffer screws experienced higher stresses, while the maximum displacements presented for the cases of less stiff materials. Nevertheless, the highest values were less than 6⋅10^–2^ mm, which were considered to be small.

### Graft Pretension

The role of the initial graft tension seems to be a controversial issue, since there is no consensus about the optimal tension. A relatively small value of graft pretension will potentially lead to continued instability, whereas a very high value is going to restrain the locomotion of the knee joint, accelerate osteoarthritis and put the graft survivability at risk (Fu et al., 1999; Beasley et al., 2005). In fact, the behavior of the graft, as the tension increases, relies strongly on the fixation device and the mechanical characteristics of the graft (Beasley et al., 2005).

A number of researchers have proposed a 44 N initial graft tension. However, this value has not been scientifically ascertained (Fu et al., 1999). In their clinical study, Yasuda et al. (1997) applied a 20, 40, and 80 N of initial tension on the hamstring tendon graft. The corresponding anterior-posterior translations were 2.1, 1.4, and 0.6 mm, respectively. It was concluded that the group with the 80 N initial tension demonstrated considerably less anterior laxity compared to the group of 20 N. On the contrary, in van Kampen et al. (1998) no considerable dissimilarities were observed concerning postoperative stability in response to different values of initial tension on the BPTB grafts. Hence, additional investigations are required for the purpose of comprehending the significance of the initial graft tension.

**FIGURE 8:**
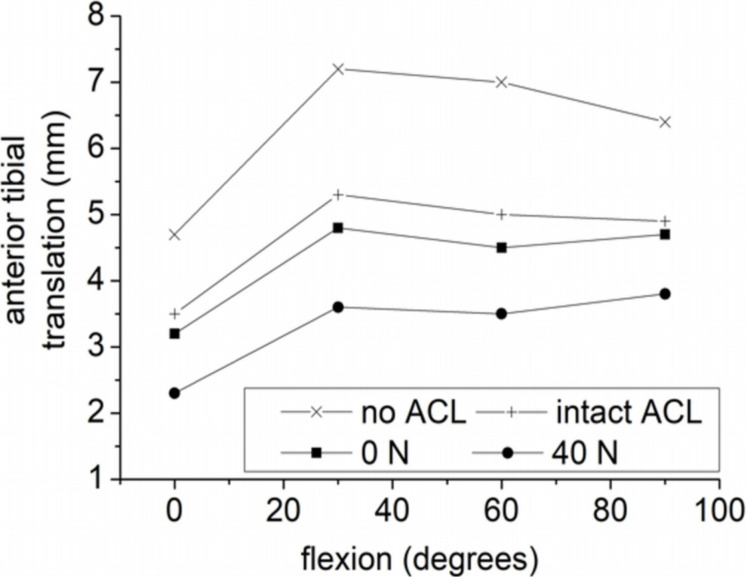
Anterior tibial translation of a deficient (no ACL) and intact ACL knee as well as via incorporating a BPTB graft with 0 and 40 N initial tension. The data for the plots were derived from the study of Suggs et al. (2003).

An indicative FE study, which was also the first one that examine the effect of initial tension of the graft, is that of Suggs et al. (2003). Since the content of this study has already been analyzed above, here only the impact of initial tension is presented. The femur was fixed, while the tibia was permitted to have free movement apart from the direction of flexion. A 134 N anterior force was applied to the tibia for flexion angles (0–90°) simulating the cases of a deficient ACL, an intact ACL and three grafts. As a characteristic example, out of the three grafts only the third one is considered here, namely the BPTB graft, with an axial modulus of 30 kN as well as the resulting anterior tibial translation based on (Suggs et al., 2003) findings. The numerical results demonstrated that ACLR has been effective, as the anterior tibial translation was restored close to its normal, thus, contributing to knee stability. As can be gleaned from Figure 8, the resulting knee kinematics for the case of zero initial graft tension restored the corresponding values of the intact ACL. However, the slightly over-constrained behavior of 0 N case study became more intense when a 40 N initial tension was applied. As pointed out by Suggs et al. (2003), this over-constraint of the knee can be a result of a combination of high graft stiffness and initial tension leading to a tight ACL substitute.

Peña et al. (2005) also examined the effect of the initial graft tension on the knee. Similar to the FE analysis of Suggs et al. (2003), an anterior tibial load equal to 134 N was applied, while the resulting anterior translation of the tibia was estimated. The values for the initial tension were 0, 20, 40, and 60 N with the flexion angle of the knee being equal to 0, 30, and 60°. In addition, a BPTB was selected for this comparative study. It should be stressed that, in contrast to Suggs et al. (2003), a 3D graft representation was utilized by incorporating a hyperelastic transversely isotropic material model which enabled the investigation of the stresses within the soft tissue. They concluded that the knee with no graft pretension could not reconstruct the healthy knee joint kinematics. This contradiction against the results of Suggs et al. (2003) was attributed to the simplification of using springs in Suggs et al. (2003) instead of 3D elements for the graft representation. Overall, Peña et al. (2005) found that the implementation of initial tension is very crucial for the ACLR success. The closest to the intact knee results were those of 60 N. Nevertheless, significant stresses were observed in postoperative healing and revascularization that can potentially cause problems. The optimum scenario was that of 40 N pretension, which is very close to the value of 44 N suggested by clinical studies mentioned above.

In Bae and Cho (2019), the stress and strain on the graft were calculated via different combinations of possible tunnel positions and four pretension forces, namely 0, 10, 20, and 30 N. The stresses were more influenced by tunnel location than pretension in most of the combinations. Thus, the optimal tunnel regions were considered. Regarding the effect of graft pretension, no effect was observed at 5 mm sites in the posterior, anterior as well as posterior proximal sites. The worst tunnel positions in femur were proved to be the proximal and anterior sites. As a consequence, it was suggested that clinicians avoid putting the tunnel start point of the femur at the proximal and anterior locations as far as possible.

Naghibi et al. (2020) calculated different fixation forces for each graft in order to restore the intact ACL kinematics. In particular, the quadriceps tendon demonstrated the higher fixation force, which was around 80 N, while the hamstring and patellar tendon grafts required around 40 N. Finally, the studies of Peña et al. (2006), Chizari et al. (2011), Kim et al. (2011), Westermann et al. (2013), Bae et al. (2016), and Tampere et al. (2019) included a single value for the pretension of the graft without investigating the effect of different values. Overall, the pretension values that have been tested in the literature regarding the FE studies are those presented in Table 5.

**TABLE 5:** FE studies which have applied an initial graft tension for ACLR.

Study	Suggs et al., 2003	Peña et al., 2005	Bae and Cho, 2019	Chizari et al., 2011	Kim et al., 2011; Westermann et al., 2013	Peña et al., 2006	Bae et al., 2016	Tampere et al., 2019
Pretension (N)	0, 40	0, 20, 40, 60	0, 10, 20, 30	5	20	40	44	50

## Conclusion

In this study, a review was presented regarding the FE modeling and simulation of the ACLR, a surgery technique which aims at restoring knee kinematics. Fundamentals of ACLR and ACL structure were presented along with a brief description on mesh generation and modeling of the knee joint geometry. Emphasis was given to the material modeling and properties of the most popular grafts for replacing the native ACL. The selected publications were analyzed within different subsections for the purpose of demonstrating the effect of each factor individually and summarizing the current clinical advancements. To this end, the evaluation via FE analysis of different surgery techniques, namely the use of single or double bundles, initial graft tensioning as well as various kinds of grafts and fixation devices, was presented.

In brief, out of the 26 reviewed papers, only 15.4% of them encompassed the patellofemoral joint, while a 23.1% simulated the double-bundle ACLR. Additionally, the inclusion of graft pretension concerned the 34.6% of the studies, whereas almost half of them (46.2%) did not include neither cartilages nor menisci. Regarding the material modeling, most of the selected papers used a linear elastic representation of the above soft tissues. Furthermore, bones were considered to be rigid bodies (65.4%), owing to their high Young’s modulus and density as compared with soft tissues, as well as either linear elastic (30.8%) or hyperelastic (3.8%). In 11.5% of the studies also linear elastic bone plugs were taken into account. The ligaments and grafts were commonly represented as hyperelastic (46.15%), linear elastic (23.1%), and nonlinear tensile springs (19.2%) materials, while some models considered grafts and ligaments as fiber reinforced materials with a hyperelastic ground matrix (19.2%).

Concerning the loading and boundary conditions, there are mainly two approaches that depend on whether the analysis focuses on evaluating the stress distribution due to different fixation device or evaluating the knee joint kinematics after ACLR. Publications related to the former applied a tensile force as a means to capture the graft tension at full extension during gait (Harrington, 1976). For the latter, researchers implemented an anterior tibial load or an internal torque at the tibia for comparing the resulting knee kinematics with those of intact or deficient ACL.

It is concluded that more comparative studies are required for the assessment of the number of bundles (one or two) needed for the optimal kinematic result. Thus far, it seems that double-bundle ACLR contributes to better rotational stability. However, no consensus exists on the graft fixation, graft selection and optimal initial graft tension. A very high tension seems to restrain the knee joint motion, while smaller values may lead to instability. For the graft description, spring elements and different material models have been explored, including linear elastic and hyperelastic models (such as neo-Hookean) as well as transversely isotropic models in order to account for the tissue’s reinforcement due to the aligned collagen fibers. Overall, the optimal result should demonstrate similar translation and rotation with the healthy knee joint.

In order to obtain realistic numerical estimates when simulating ACLR, several important aspects should be considered:

• Accurate geometrical representation of the main structural elements of the knee joint, i.e., bones, cartilages, menisci, ligaments, and grafts.
• Proper choice of material models in order to describe accurately the mechanical behavior of the knee tissues.
• The application of realistic loading and boundary conditions.

• Realistic step-by-step simulation of the surgical procedure according to the clinical practice.

The incorporation of the associated muscles in a FE model of the knee joint and the application of accurate muscle forces is an improvement toward a more realistic modeling approach (Ali et al., 2016). This is, however, a challenging task, especially if the 3D representation of the muscle tissues is required and their contractile behavior has to be described (Spyrou and Aravas, 2012). Further advancements in FE knee joint modeling should also be related to the multiscale material modeling of the tissues. This will contribute to the investigation of the biomechanical behavior from the level of the joint to the level of a tissue cell, following modeling frameworks recently proposed for example regarding cartilage and muscle tissue (Spyrou et al., 2019; Tanska et al., 2020). Finally, bearing in mind the current advances in FSI numerical modeling, the role of the synovial fluid in the mechanical response of the knee joint can be investigated following an FSI modeling approach (Hron and Mádlík, 2007). A key advantage of FSI modeling is that one solution procedure can be implemented to simulate both structural, fluid and FSI simultaneously (Dale and Holdø, 2004).

In a nutshell, more effort should be put on FE studies, given the relevant progress in both FE modeling and ACLR. Numerical simulations, based on FE, can make predictions about the developed stresses in biological tissues and evaluate what-if scenarios *in silico*, without clinical intervention. As a consequence, accurate numerical modeling along with interdisciplinary cooperation could arguably constitute a valuable tool in the amelioration of the ACLR surgeries. It is expected that this review study will contribute to more systematic research regarding the numerical evaluation of ACLR, which may result in the improving of the existing surgical techniques.

## Study Limitations and Strengths

The main limitation of this investigation is the relatively small number of the publications that were taken into account. This is attributed mainly to the intrinsic difficulty and complexity of the surgery itself and the various factors involving in it, which require a strong theoretical background on both clinical and FE field.

The major strength and innovation of the present comprehensive review, however, is that provides the current progress regarding the different surgery techniques in both FE and clinical level for the first time, at least to authors’ knowledge. Consequently, it presents a complete guide to anyone who wants to deal numerically with this complex problem and evaluate hypotheses *in silico* related to the planning, assessment and improvement of ACLR.

## Author Contributions

LB, DT, and LS contributed to the conception and design of the study. LB and DS wrote the first draft of the manuscript. DT, KM, and LS supervised the manuscript. All authors contributed to manuscript revision and approved the submitted version.

## Conflict of Interest

The authors declare that the research was conducted in the absence of any commercial or financial relationships that could be construed as a potential conflict of interest.

## Abbreviations

ACLR: anterior cruciate ligament reconstruction
BPTB: bone-patellar tendon-bone.
